# The natural course of shoulder instability and treatment trends: a systematic review

**DOI:** 10.1007/s10195-016-0424-9

**Published:** 2016-08-17

**Authors:** W. Eljabu, H. M. Klinger, M. von Knoch

**Affiliations:** 1 Department of Traumatology and Hand Surgery, Klinikum Bremerhaven Reinkenheide, Postbrookstr. 103, 27574 Bremerhaven, Germany; 2 Department of Orthopaedics, Göttingen University Hospital, Robert-Koch-Str. 40, 37075 Göttingen, Germany; 3 Department of Orthopaedics and Shoulder Surgery, Osterholz County Hospital, Am Krankenhaus 4, 27711 Osterholz-Scharmbeck, Germany

**Keywords:** Natural history, Natural course, Shoulder instability, Shoulder dislocation, Regression to the mean, AMQPP assessment tool

## Abstract

**Background:**

The natural course of shoulder instability is still not entirely clear. We aimed in this review to analyse the current scientific evidence of the natural history of shoulder instability.

**Materials and methods:**

A systematic review of the English literature was performed using the PubMED database throughout January 2014. This review was guided, conducted and reported according to PRISMA criteria. The criteria for inclusion in the study were (1) the article was written in English, (2) the level of evidence was 1–4, (3) the article was available in full text, (4) the article investigated the natural history or course of shoulder instability, the outcome of non-operative management, or the regression of the shoulder symptoms to the mean. The methodological quality of each included study was individually assessed using a newly developed general assessment tool—Assessing the Methodological Quality of Published Papers (AMQPP).

**Results:**

Eight articles related to shoulder instability met the inclusion criteria. Four papers were considered high-quality studies (evidence level 1 and 2). One paper assessed the natural history and the natural course of shoulder instability directly. The other studies indirectly assessed the natural history by studying non-operative and operative therapy trends. We found no articles which clearly referred to the role of ‘regression to the mean’.

**Conclusion:**

Following the natural history and the implementation of standardised non-operative treatment programmes are an effective therapy and superior to surgery in many cases. However, primary acute shoulder dislocation in young active individuals partaking in demanding physical activities could benefit from early surgical intervention. The AMQPP score works as a quick quality-checking tool which helps researchers to identify the key points in each paper and reach a decision regarding the eligibility of the paper more easily. The AMQPP scoring system is still open for further development and expansion.

*Level of evidence* Level IV.

## Introduction

Although shoulder dislocation is a common condition, the natural history, aetiology, associated pathology, prognosis and definitive treatment are still not entirely clear [1]. Shoulder instability can result from traumatic and atraumatic causes, and can be classified as anterior, posterior or inferior instability. In contrast to traumatic shoulder dislocation, which is a frequent injury in young active patients, the onset of multidirectional instability is never the result of trauma alone. The aetiology here is probably multifactorial, depending for example on labral, ligamentous or collagenous abnormalities and impaired muscular control [2]. The subjective symptoms are a heavy feeling in the shoulder girdle, stiffness, mild pain, and a feeling of instability when lifting objects [3]. Detailed evaluation of the risks and identification of the factors related to shoulder dislocation development would make it easier to identify at-risk individuals and potentially guide successful preventive and therapeutic strategies. Therefore, we systematically reviewed the literature to analyse the natural, clinical and anatomical progression of shoulder instability and determine the current status of scientific knowledge about this condition and the therapies most frequently applied.

## Materials and methods

We performed a systematic review of the available medical literature in the English language using the US National Library of Medicine/National Institutes of Health (PubMed) bank data. The review was conducted and reported according to the protocol outlined by PRISMA (Preferred Reporting Items for Systematic Review and Meta-Analyses) [4].

### Identification and selection of the literature

**Fig. 1:**
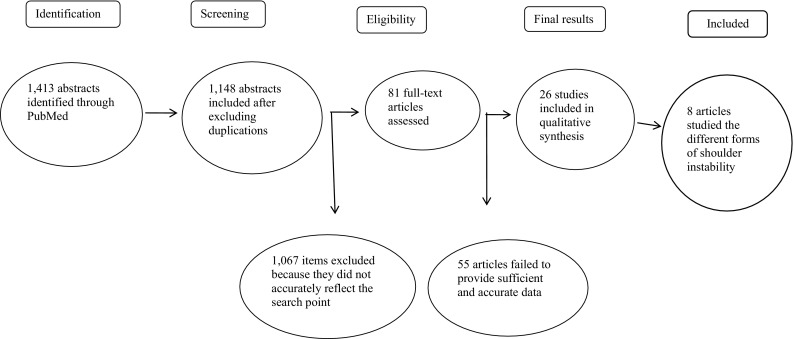
Diagram of screening process

The search for eligible literature was independently performed by the first author in January 2014, beginning with a generic search strategy to find studies on shoulder disorders in general. It was then refined with a subject-specific strategy to identify studies addressing the shoulder and its natural history, non-operative therapy and its relationship to regression to the mean (Fig. 1). The final results of the search included articles related to shoulder dislocation, shoulder instability, shoulder luxation and multidirectional instability which were selected for further review together with other articles which covered various shoulder problems [5]. Regression to the mean is a statistical phenomenon that occurs whenever there is a non-random sample from a population with two measures that are imperfectly correlated. To avoid incorrect inferences, regression toward the mean should be considered when designing scientific experiments and interpreting data. All relevant article titles and abstracts were independently screened and reviewed applying the inclusion/exclusion criteria (see below). Full-text articles were retrieved if the abstract provided insufficient information to establish eligibility. However, all articles that passed the first eligibility screening were fully read and assessed. We also reviewed the bibliographies of the included studies in order to seek additional relevant publications that were not identified in the computerised search.

### Selection criteria

The criteria for inclusion in the study were (1) the article was written in English, (2) the level of evidence was 1–4, (3) the article was available in full text, (4) the article investigated the natural history or course of shoulder disorders, the outcome of non-operative management, or the regression of the shoulder symptoms to the mean.

Exclusion criteria included (1) basic science, (2) animal models, cadaver studies or studies of an asymptomatic population, (3) biomechanical studies not reporting clinical outcomes, (4) studies reporting only imaging (X-ray, ultrasound, computed tomography or magnetic resonance imaging with no clinical assessment), and (5) systematic reviews. All criteria were applied independently. In case of disagreement, a consensus method was used to discuss and resolve the disagreement between the authors.

### Quality assessment

The methodological quality of each study was assessed using a newly developed assessment tool based on Greenhalgh’s article ‘Assessing the Methodological Quality of Published Papers’, which we abbreviated as AMQPP [6]. Because of the structural and functional varieties of the included articles, it was a difficult task to find a single existing assessment method which could be applied to all papers. Therefore, the authors created the AMQPP assessment tool (Table 1) to evaluate the essential requirements for high-quality papers. The maximum score on the AMQPP tool criteria list is 6. The total score is counted from all the criteria that score ‘yes’. ‘No’ and ‘unclear’ score no points. Based on our observations, we decided that a minimal score of 4 out of 6 is necessary for any paper to be considered. These four points have to match the first four questions of the AMQPP tool. The AMQPP assessment tool was linked with the level of evidence rating.

**Table 1:** AMQPP assessment tool

AMQPP	Yes	No	Not clear
1. Is the study original?			
2. Does the study make clear what it is about? (hypothesis clearly stated, subject recruited, inclusion and exclusion criteria, circumstances)			
3. Is the design of the study sensible? (What specific intervention or other manoeuver was considered and compared? How was the outcome measured?)			
4. Does the study deal with preliminary statistical questions? (the size of the sample, the duration of follow-up, the completeness of follow-up)			
5. Does the study avoid or minimise systematic bias?			
6. Was assessment blind? (Did the people who assessed the outcome know which group the patient they were assessing was allocated to?)			

### Data extraction

The first author independently extracted data from the selected studies on the study population, study design, hypothesis, treatments, outcomes and summary of results (Table 2). The authors (MK and MV) reviewed and confirmed the abstracted results of the first author. During the review, the authors (MK and MV) were blind to the initial abstracted results.

**Table 2:** Summary of the included papers

Author	Sample size	Study design	Hypothesis	Conclusion
Hovelius et al. [10]	255 patients	Prognostic level 1 prospective >80 % follow-up	The effectiveness of non-operative treatment (immobilisation) of primary anterior shoulder dislocation in patients up to the age of 40	Half of the primary anterior shoulder dislocations that had been treated non-operatively in patients aged between 12 and 25 years had not recurred and had become stable over time
Maquieira et al. [7]	14 patients	Therapeutic level 4 case series	To observe patients with large (>5 mm), displaced (>2 mm) non-operatively treated anterior glenoid rim fractures	Traumatic anterior shoulder dislocation with a large, displaced rim fracture can be treated successfully without surgery, provided the glenohumeral joint is concentrically reduced
Buss et al. [12]	30 patients	Therapeutic level 4 case series	To determine if in-season athletes can be returned to their sports quickly and effectively after non-operative treatment of an anterior instability episode	Most young athletes were able to return to their sport and complete their seasons after an episode of anterior shoulder instability, although 37 % experienced at least one additional episode of instability during the season. However, the long-term effect of conservative treatment was not determined in this study
Bottoni et al. [13]	24 patients	Therapeutic level 1 prospective randomized clinical trial	To determine whether early arthroscopic treatment for shoulder dislocation can result in a lower recurrence rate than non-operative treatment	Arthroscopic stabilisation (Bankart repair) of traumatic, first-time anterior shoulder dislocations is effective and significantly reduces the recurrence rate when compared with conventional non-operative therapy
Kiss et al. [11]	59 patients	Therapeutic level 3 retrospective comparative	To assess the results of the non-operative treatment of patients who were referred to a tertiary unit with a diagnosis of multidirectional instability and were treated with a rehabilitation programme with no planned surgical intervention	Conservatively treated patients with multidirectional shoulder instability developed more confidence in everyday activities and had a better understanding of their shoulder condition despite persisting clinical signs of laxity and instability. Patients who had undergone previous shoulder surgery, sustained a work-related injury or had psychological problems were unlikely to benefit from the rehabilitation programme
Kuroda et al. [8]	341 patients	Prognostic level 4 case series	To follow the natural course of atraumatic shoulder instability	Spontaneous recovery occurred in 50 cases. The incidence of spontaneous recovery in the group that discontinued overhead sports was 8.7 times greater than in the group that continued to play overhead sports
Wintzell et al. [15]	30 patients	Therapeutic level 1 prospective randomized	The clinical value and the treatment results of arthroscopic lavage on patients with traumatic primary anterior shoulder dislocation	Arthroscopic lavage significantly reduced the risk of recurrent anterior shoulder dislocation in young patients when compared with non-operative treatment
Arciero et al. [14]	36 patients	Therapeutic level 2 prospective comparative	To study arthroscopic Bankart repair versus non-operative treatment for acute, initial anterior shoulder dislocation	Arthroscopic Bankart repair significantly reduced the rate of recurrence in young athletes compared to non-operative measures

## Results

The PubMed search resulted in 1,413 citations. Of these, 265 duplicates were removed, leaving 1,148 titles with abstracts to review. After the first screening, the full-text articles of 26 potentially eligible citations were retrieved. Eight studies dealing specifically with shoulder instability were finally included in this systematic review after 18 articles on other shoulder disorders had been excluded (Fig. 1).

None of the studies clearly identified the role of regression to the mean in shoulder diseases. Using the original description of ‘regression to the mean’ in the search process, we found no articles which used this specific term in connection to shoulder instability. We also found no articles which dealt with the concept of ‘regression to the mean’ using similar terms. The quality assessment tool (AMQPP) revealed three articles which scored the full mark of 6. One article scored 5, and the remaining four scored 4 out of 6 because the papers did not specify whether the assessors were blind to the patient’s treatment programme, and there was no clear action to minimise the systematic bias. This result correlated well with the level of evidence rating of the included articles.

A summary of the characteristics of each study is presented in Table 2. The samples in the included studies ranged from 14 [7] to 341 [8] patients. The table contains four high-quality studies, three of which had level 1 evidence according to the Oxford Centre for Evidence-Based Medicine [9]. The remaining four articles were case series or case−control studies (level 3 and 4). Of these eight articles, six studies were designed to be therapeutic and two were prognostic. All the studies had directly or indirectly investigated the natural history of shoulder dislocation and evaluated the effect of patient characteristics on the outcome of the disease and on certain therapeutic measures.

Most of the authors were in favour of allowing time for spontaneous recovery when possible and for conservative management in general. In their prognostic level I study of 255 patients (257 shoulders), Hovelius et al. [10] found that half of the primary anterior shoulder dislocations in their study which had been treated non-operatively in patients aged 12–25 years had not recurred and had become stable over time. Sixty-two shoulders (27 %) underwent surgery because of instability. The remaining shoulders (22 %) were also classified as having recurrent dislocation or subluxation but did not undergo surgery. Interestingly, there was no significant correlation between activity levels and recurrence rate, and the risk for recurrence was in fact higher in the group of patients who participated in no sports at all. Furthermore, a small fracture of the glenoid rim or an impression fracture of the humeral head at the time of primary dislocation did not seem to influence the recurrence rate. The disabilities of arm, shoulder and hand (DASH) scores for the shoulders classified as non-recurrent, stable over time, and surgically stabilised were similar; those with persistent recurrent dislocations (7.9 %) fared worse compared with other groups. On average, women scored worse than men did and there was no identifiable difference in the DASH scores when the dominant and non-dominant shoulders were compared. Kiss et al. [11] studied 59 patients (37 female, 24 male) with 84 previously symptomatic—62 shoulders had received no previous surgical treatment while 22 had failed to respond to surgical treatment before the rehabilitation programme. The average follow-up was 3.7 (1–10) years. They found that conservatively treated patients with multidirectional shoulder instability developed more confidence in everyday activities and had a better understanding of their shoulder condition despite persisting clinical signs of laxity and instability. A higher proportion of persisting signs of instability was observed among previously operated shoulders. Younger patients and males did slightly better. Shoulders with instability in all three directions had better results compared to shoulders with postero-inferior instability. Patients who had undergone previous shoulder surgery, sustained a work-related injury or had psychological problems were less likely to benefit from the rehabilitation programme. According to the Rowe score, the proportion of shoulders with fair or poor function amongst the shoulders that had been treated surgically before rehabilitation was considerably higher. The study by Maquieira et al. [7] of a cohort of 14 patients with a mean follow-up of 5.6 years showed that non-operative treatment of traumatic anterior dislocation of the shoulder, associated with a large displaced antero-inferior glenoid rim fracture led to a stable, functional shoulder and high patient satisfaction. The mean Constant score and subjective shoulder values were 98 % (range 90–100 %) and 97 % (range 90–100 %), respectively. There were no re-dislocations or subluxations, and the apprehension test was negative. The size of the fragment was relevant only if the fracture length was more than half the greatest diameter of the glenoid. All fragments healed with an average intra-articular step of 3.0 mm (0.5–11). Development of osteoarthritis was not a clinical problem, as only three shoulders with mild to moderate radiological osteoarthritis had a tendency to anterior subluxation, but they were asymptomatic. Kuroda et al. [8] followed the natural course of atraumatic shoulder instability in 341 patients (573 shoulders) for >3 years. There were 467 cases of multidirectional shoulder instability, 49 cases of voluntary dislocation (<1 %) and 56 cases of habitual dislocation. Spontaneous recovery was documented in 43 cases of loose shoulder (9 %) and in seven cases of habitual dislocation (12.5 %) (28 women and 22 men with an average age of 20.1 years); it was mainly in patients who made changes in their sport activities by discontinuing overhead sports (volleyball, handball or baseball) and contact sports. In 31 cases there was a change in shoulder instability with no shift of disorder. However, in 50 (8.7 %) cases there was a shift of disorder between multidirectional instability, voluntary dislocation, habitual dislocation, and sustained subluxation. There was no spontaneous recovery in 18 cases who exhibited general joint laxity and Endo type III loose shoulder. Patients were treated conservatively for at least 2 years before any surgery was considered. Buss et al. [12] treated 30 young athletes (contact sports) with anterior shoulder instability over a 2-year period. Interestingly, 27 (90 %) athletes showed spontaneous recovery and were able to return to their sport and complete their season after an episode of anterior shoulder instability, although 37 % experienced at least one additional episode of instability during the season. All patients were treated with physical therapy and, if appropriate, fitted with a brace on return to play (19 %) to provide more stability. The athletes subjectively reported an improved sense of stability compared to playing without a supplemental device. The average number of days missed in sports due to primary and recurrent episodes was 10.2 (range 0–30 days). However, Bottoni et al. [13], Arciero et al. [14] and Wintzell et al. [15] emphasised the role of therapeutic measures (arthroscopic lavage and stabilisation) as an effective method to reduce the recurrence rate. This treatment resulted in a lower recurrence rate and an improved overall outcome in comparison with traditional non-operative treatment, mainly of acute shoulder dislocation in young patients (<25 years) who were known to have a high recurrence rate. A prospective randomised study was performed on 30 consecutive patients with traumatic primary anterior shoulder dislocation to compare the results of arthroscopic lavage with those of conventional non-operative treatment [15]. The patients were between 18 and 30 years of age and had no history of shoulder problems. At the 2-year follow-up, 20 % of the patients in the lavage group had a re-dislocated shoulder compared with 60 % of patients in the non-operative group. However, functional outcome according to the Constant and Rowe shoulder scores did not reveal any significant differences between the groups. All 30 patients worked full time with an equal distribution between the two groups. The amount of sick leave taken was similar in both groups with an average of 15 days. The mechanism by which arthroscopic lavage reduced the rate of recurrence was not stated in this study. In a prospective study, Arciero et al. [14] evaluated non-operative treatment versus arthroscopic Bankart suture repair for acute, initial dislocation of the shoulder in 36 young athletes. Group I patients were immobilised for 1 month followed by rehabilitation and full activity at 4 months. Group II patients underwent arthroscopic Bankart repair followed by the same protocol as Group I. Recurrent instability developed in 80 % of Group I patients (12 patients); seven of these patients required open Bankart repair. Recurrence occurred during a collision sport in eight patients, and during a limited contact sport in four patients. Eighty-six percent of Group II patients (18 patients) had no recurrent instability at the last follow-up (after an average of 32 months) and only one patient required a subsequent open Bankart repair.

## Discussion

The natural course of shoulder instability is still obscure due to the shortage of published studies. Better understanding of the various phases of the disease and identification of factors that lead to symptoms is vital in order to establish guidelines for management and treatment. In view of the lack of sufficient material dealing with the natural history of shoulder instability, a high-quality systematic review of this topic seems a difficult undertaking.

The level of evidence of the included papers varied. Case series studies and retrospective uncontrolled studies could not be ignored as they provided a rich foundation for an understanding of the mainstream literature. Together with higher-quality papers, case series and uncontrolled studies were crucial for creating a complete picture of the frequency of shoulder instability and therapeutic trends. As a consequence, the purpose and methodology of the included articles were different, and it was difficult to find a single existing quality assessment tool which could be applied to all papers. During the last 15 years, the Coleman methodology score has been used to assess the methodological quality of the literature [16, 17]. The initial purpose of this scoring system was to analyse the quality of studies reporting surgical procedures on the patellar and Achilles tendons. The criteria not only take into account the study design and methodology but also assess the quality of the outcome. However, Coleman’s scoring system is not only complex, it undermines the value of retrospective studies and case series because it does not take the retrospective nature of a study into consideration and includes the sample size criteria, which further reduces the score. Hence, the maximum score that can be obtained by a retrospective study is only 65 [18]. Furthermore, the Coleman methodology score in fact assesses the quality of reporting, rather than the quality of the study. Therefore, a high-quality study that is poorly written would receive a low score [19]. We therefore wanted to create an assessment tool that would be simple and fairer to all kinds of studies. We used Greenhalgh’s article [6] as a guideline, developed the AMQPP assessment tool and applied its criteria to all the included papers (Table 1). Using this tool we considered six essential questions that should form the basis of every study. We assessed the method and design section of each paper, what the study was about, whether the systematic bias was avoided or minimised, and whether the sample was large enough and the study continued for a sufficient length of time to make the results credible. We believe that no scoring domain should have greater weight than any other, despite the risk of jeopardising the objectivity of each domain. However, a minimal score of 4 out of 6 is necessary for any paper to be considered. These four points have to match the first four questions of the AMQPP tool (Table 1). The AMQPP scoring system is intended as a quick quality-checking tool which helps researchers identify the key points in each paper and reach a decision regarding the eligibility of the paper more easily. This scoring system could undoubtedly be improved further in the future.

Regression to the mean is a statistical ubiquitous phenomenon in repeated data and should always be considered as a possible cause of an observed change. Its effect can be alleviated through better study design and the use of suitable statistical methods [20]. Many patients who were operated on at an early stage certainly had a good recovery, but the studies provided no proof that this recovery was indeed the result of surgery and not just a result of natural history and time. Unfortunately, in this review, no clear evidence was found that the studies had investigated the role of regression to the mean of shoulder problems.

Recurrent instability and deficits in shoulder function are common after primary shoulder dislocation. The rate of re-dislocation after an initial traumatic shoulder dislocation varied in different reports. The level of activity resumed by a patient after an initial dislocation may determine the risk of re-injury [13]. In young active patients the recurrence rate of shoulder instability is still high regardless of treatment [14], with females having a much lower risk [21]. No substantial difference was reported between dominant and non-dominant extremities. Most first-time dislocations should ideally be treated non-operatively [22]. A high incidence of spontaneous recovery was reported in patients who had greater awareness of their condition and who modified their sport activities [8]. Hovelius et al. [10] found that immobilisation with the arm tied to the torso for 3–4 weeks following primary dislocation did not change the prognosis compared with immediate mobilisation, which may represent the true ‘natural history’, as no difference could be shown between the two treatment groups in their study. In the case of associated fracture (glenoid fragments), the size and displacement of the fragment are important when deciding on treatment. Itoi et al. [23] recommended immobilisation of the shoulder in ten degrees of external rotation for 3 weeks after glenohumeral dislocation associated with a large, displaced glenoid rim fracture. However, open reduction with internal fixation was recommended when the fragment was displaced by >10 mm and corresponded to one-quarter of the glenoid surface [24]. In general, patients who require a full return to a high level of fitness and function within a short period, as well as those who are at a higher risk of re-injury, might need to undergo surgical stabilisation of a primary shoulder dislocation [25]. Bottoni et al. [13] evaluated the use of an arthroscopically inserted bio-absorbable tack to repair capsulolabral injury associated with acute shoulder dislocation in comparison with traditional non-operative treatment. The ability of surgery to reverse the natural history of this problem suggests that restoring the capsulolabral complex back to its anatomical position can lead to normal function without instability. However, there is no conclusive evidence to indicate whether operative stabilisation or conservative rehabilitation is more effective in the treatment of other patients or injury types. Moderate to severe osteoarthritis and arthropathy are relatively uncommon (9 %) 10 years after a shoulder dislocation [10]. Moreover, the degree of osteoarthritis did not seem to be related to the number of dislocations or whether the patient had undergone surgical stabilisation.

Any review is limited by the quality of the studies contained therein. Several methods by which the studies were carried out may have had the potential to inflict bias on the results. Regression to the mean was not used in the majority of the studies. Moreover, the level of activity among patients was not well defined, nor constantly measured by validated scales. There were also some differences among the conservative treatment protocols, duration of immobilisation either after acute injuries or after surgery, and the length of the follow-up period.

In summary, there is sufficient evidence to assume that symptoms and physical findings alone are reliable enough to predict the clinical status of shoulder instability. The development of symptoms is directly correlated to anatomical deterioration. Different forms of shoulder dislocation have been successfully treated without surgery and spontaneous recovery to normal levels of function has been achieved. Following the natural history and the implementation of standardised non-operative treatment programmes of shoulder dislocation results in a more favourable outcome than surgical intervention in many cases. However, primary acute shoulder dislocation in young active individuals participating in demanding physical activities could benefit from early surgical intervention. The AMQPP score system is simple and straight forward. It works as a quick quality-checking tool which helps researchers to identify the key points of each paper and reach a decision regarding its eligibility more easily. The natural history of shoulder instability still needs more research and requires greater attention from the field of orthopaedics.
